# Mechanisms regulating nutrition-dependent developmental plasticity through organ-specific effects in insects

**DOI:** 10.3389/fphys.2013.00263

**Published:** 2013-09-26

**Authors:** Takashi Koyama, Cláudia C. Mendes, Christen K. Mirth

**Affiliations:** Development, Evolution and the Environment Laboratory, Instituto Gulbenkian de CiênciaOeiras, Portugal

**Keywords:** IIS/TOR signaling, nutritional plasticity, body/organ size, relative organ growth, organ-specific sensitivity, ecdysone, juvenile hormone

## Abstract

Nutrition, via the insulin/insulin-like growth factor (IIS)/Target of Rapamycin (TOR) signaling pathway, can provide a strong molding force for determining animal size and shape. For instance, nutrition induces a disproportionate increase in the size of male horns in dung and rhinoceros beetles, or mandibles in staghorn or horned flour beetles, relative to body size. In these species, well-fed male larvae produce adults with greatly enlarged horns or mandibles, whereas males that are starved or poorly fed as larvae bear much more modest appendages. Changes in IIS/TOR signaling plays a key role in appendage development by regulating growth in the horn and mandible primordia. In contrast, changes in the IIS/TOR pathway produce minimal effects on the size of other adult structures, such as the male genitalia in fruit flies and dung beetles. The horn, mandible and genitalia illustrate that although all tissues are exposed to the same hormonal environment within the larval body, the extent to which insulin can induce growth is organ specific. In addition, the IIS/TOR pathway affects body size and shape by controlling production of metamorphic hormones important for regulating developmental timing, like the steroid molting hormone ecdysone and sesquiterpenoid hormone juvenile hormone. In this review, we discuss recent results from *Drosophila* and other insects that highlight mechanisms allowing tissues to differ in their sensitivity to IIS/TOR and the potential consequences of these differences on body size and shape.

Much of the diversity seen across insect species is generated by changes in organ size and shape relative to the whole body (Emlen and Nijhout, 2000; Shingleton et al., 2008). Even within a species, we find dramatic examples where the size of an organ changes disproportionately with increasing body size. In dung beetles, the relative size of male horns, used in courtship battles to gain access to females, varies disproportionately with body size (Arrow, 1951; Emlen, 1994, 1997a). Small-bodied males have very small horns similar to those of females. In contrast, above a threshold body size, males develop much larger horns (Emlen, 1997b). Although differential growth between the organs and the whole body appears more dramatic in polyphenic insects like dung beetles, similar phenomena occur on a subtler scale in all insects to shape the final adult form (Shingleton et al., 2007, 2008).

Although we are beginning to elucidate the mechanisms regulating whole body size, understanding differences in the relative growth of organs poses a new challenge. The regulation of the relative size of organs must, in part, be determined by the mechanisms that govern body size. However, because organs differ in their scaling relationships with body size, there must also be additional organ-specific mechanisms for growth. Furthermore, because organs do not grow at the same rate throughout development, we expect the organ's response to a given growth factor to change over developmental time.

Here, we review the findings over the past fifteen years that describe how body size changes in response to an environmental cue, nutrition, through the action of the insulin/insulin-like growth factor signaling (IIS)/target of rapamycin (TOR) signaling pathway. Further, we will discuss how the IIS/TOR pathway affects endocrine tissues to regulate the production of two metamorphic hormones: the steroid molting hormone ecdysone and the sesquiterpenoid hormone juvenile hormone (JH). Finally, we present our hypothesis explaining how the interplay between the IIS/TOR pathway and the regulation of tissue growth by ecdysone and JH might act to mould organism shape. This hypothesis serves as a framework for evolutionary/developmental studies of nutrition-based phenotypic plasticity.

## Nutrition-dependent signaling via the IIS/TOR pathway

Body size is a function of larval nutrition in insects. Once larvae initiate metamorphosis, adult body size becomes fixed, as insects do not feed during the pupal stages and the sclerotized outer skeleton of the adult body does not permit further growth. In insects that undergo complete metamorphosis (holometabolous insects), many adult organs develop inside the larval body as imaginal primordia or discs. As the larva eats, these imaginal tissues grow and respond to the same cues that control whole body growth.

**Figure 1:**
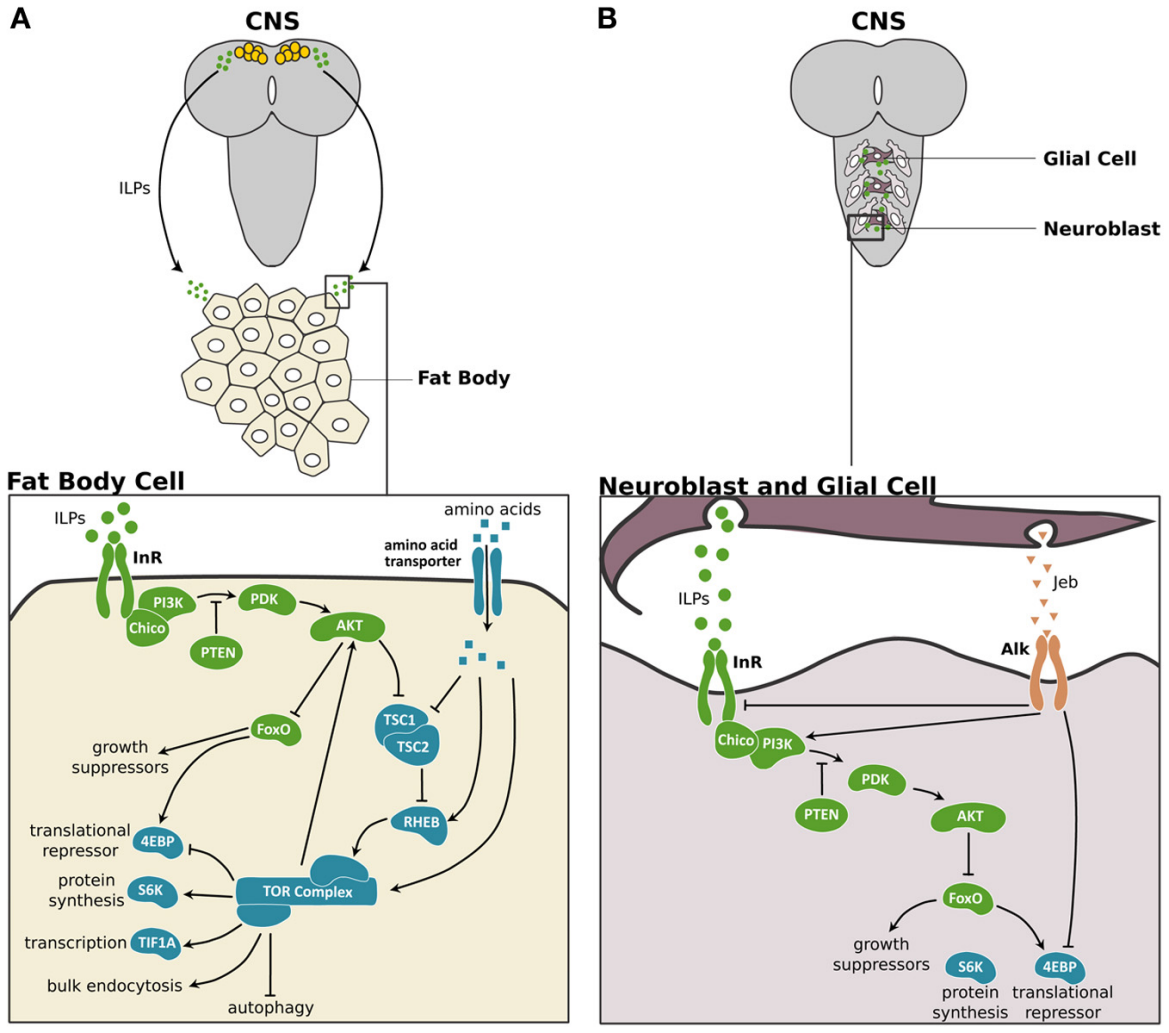
**The IIS/TOR signaling pathway in fat body and the central nervous system of *Drosophila***. **(A)** In *Drosophila*, three of the eight ILPs, ILP2, ILP3, and ILP5 are expressed in a set of neurosecretory cells in the central nervous system (CNS) (Brogiolo et al., 2001; Ikeya et al., 2002). These ILPs activate insulin/insulin-like growth factor signaling (IIS) in peripheral tissues like the fat body cells. In the fat body, the ILPs bind to and activate the Insulin Receptor (InR), which in turn activates Chico, the insulin receptor substrate. This activation initiates a phosphokinase signal transduction cascade that involves phosphatidylinositide 3-kinase (PI3K), the phosphoinositide-dependent protein kinase 1 (PDK) and the protein kinase Akt (Sarbassov et al., 2005). The phosphatase and tensin homolog (PTEN) catalysis the reverse reaction promoted by PI3K, thereby inhibiting the IIS pathway (Goberdhan et al., 1999; Gao et al., 2000). When activated, Akt promotes cell growth by inhibiting the transcription factor Forkhead Box class O (FoxO) (Junger et al., 2003), which activates the 4E-Binding Protein (4EBP), a translational repressor (Miron and Sonenberg, 2001). Furthermore, Akt suppresses the negative regulators of the Target of Rapamycin (TOR) pathway, Tuberous Sclerosis Complex 1 and 2 (TSC1/2) (Gao and Pan, 2001; Gao et al., 2002). TOR activity itself is enhanced by high concentrations of intracellular amino acids (Gao et al., 2002) via the Ras Homolog Enhanced in Brain (RHEB; Garami et al., 2003). TOR activity promotes cell growth by enhancing translation and ribosome biogenesis through inhibition of the 4EBP and activation of the ribosomal protein S6 kinase (S6K). TOR also stimulates rRNA synthesis by activating the Transcriptional Intermediary Factor 1A (TIF-1A) (Hietakangas and Cohen, 2007). Lastly, TOR promotes bulk endocytosis and inhibits autophagy. TOR feeds back on the IIS pathway by regulating Akt in a cell autonomous manner (Hietakangas and Cohen, 2007). **(B)** In the CNS two ILPs, ILP3 and ILP6, are secreted by surface glia and activate the IIS pathway in the neuroblasts to regulate the growth of this tissue (Chell and Brand, 2010; Sousa-Nunes et al., 2011). The anaplastic lymphoma kinase (Alk) and its ligand, Jelly belly (Jeb) promote growth of neuroblasts in starved larvae (Cheng et al., 2011). Alk promotes CNS growth in starved conditions by suppressing InR and directly stimulating PI3K activity. IIS components are shown in green, TOR pathway components are shown in blue and Jeb/Alk components are shown in orange.

In organisms ranging from insects to humans, the IIS/TOR pathway regulates growth in response to nutrition (Figure 1). In the fruit fly, *Drosophila melanogaster*, rich nutritional environments cause a set of neurosecretory cells in the brain, the insulin producing cells (IPCs), to produce and secrete three insulin-like peptides (ILPs), ILP2, ILP3, and ILP5 (Brogiolo et al., 2001; Ikeya et al., 2002). Starvation represses both the synthesis and secretion of these ILPs (Brogiolo et al., 2001; Ikeya et al., 2002; Géminard et al., 2009). Further, ablating the IPCs genocopies the effects of starvation (Rulifson et al., 2002; Broughton et al., 2005), suggesting that the ILPs produced in the IPCs are major mediators of nutrition-dependent growth.

Although the functions of the IIS/TOR pathway are conserved across insects, the number of ILPs varies greatly between species. There are eight ILPs in *Drosophila* and the mosquito, *Aedes aegypti* (Brogiolo et al., 2001; Riehle et al., 2006; Colombani et al., 2012; Garelli et al., 2012), two in the honeybee, *Apis mellifera* (Corona et al., 2007), and thirty two ILPs in the silkworm, *Bombyx mori* (Iwami, 2000).

Nutrition-dependent ILP production in the IPCs is thought to regulate most growth. Nevertheless, there are several additional sources of ILPs important for stage- or tissue-specific growth. For instance, the mid gut, imaginal discs, ventral nerve cord, and salivary glands also express ILPs in *Drosophila* (Brogiolo et al., 2001), and these ILPs are thought to have systemic effects on growth. In the *Drosophila* central nervous system (CNS), growth of the neuroblasts results from local ILP production in the glia, and not from the IPCs (Chell and Brand, 2010; Sousa-Nunes et al., 2011) (Figure 1B). Furthermore, when larvae stop feeding at the onset of metamorphosis, tissue growth is sustained through the secretion of ILP6 primarily by the fat body (Okamoto et al., 2009b; Slaidina et al., 2009). Thus, the pool of ILPs that mediates growth is diverse, both in its spatial and temporal expression.

Irrespective of the source, all ILPs are thought to bind to the Insulin Receptor (InR). Dipterans and lepidopterans have one InR (Graf et al., 1997; Tatar et al., 2001; Koyama et al., 2008), whereas hymenopterans have two (Corona et al., 2007; Lu and Pietrantonio, 2011). By binding to InR, ILPs activate a series of kinases such as Akt (Sarbassov et al., 2005) to promote growth (Figure 1; for more details see Nijhout et al., 2013).

The insulin pathway interacts with two additional nutrition sensitive pathways, the TOR and AMP-activated protein kinase (AMPK) pathways, to regulate growth. The TOR pathway responds directly to intracellular amino acid concentrations to regulate Akt in a cell autonomous manner (Hietakangas and Cohen, 2007) (Figure 1A). In addition, insulin signaling itself acts through Akt to suppress the negative regulators of TOR signaling, Tuberous Sclerosis Complex 1 and 2 (TSC1/2) (Gao and Pan, 2001; Gao et al., 2002). Because these two pathways converge in function, they are often referred to as the IIS/TOR pathway.

The AMPK pathway senses energy levels in the cell by responding to intracellular adenosine nucleotide levels to regulate growth and metabolism in *Drosophila* larvae (Braco et al., 2012; Mihaylova and Shaw, 2012). In *Drosophila* larvae, blocking AMPK signaling appears to regulate growth by affecting contraction of the visceral muscle, thereby interfering with gut function (Bland et al., 2010). In mammals, AMPK signaling interacts with IIS/TOR by regulating TSC1/2 (Mihaylova and Shaw, 2012). Thus, AMPK is also considered part of this signaling network, although a direct molecular link has yet to be established in *Drosophila*.

In response to IIS/TOR signaling, Akt acts on a series of downstream targets, thereby inducing organ growth. For example, Akt indirectly activates S6 kinase (S6K), which enhances ribosome synthesis (Miron et al., 2003) and cell growth (Montagne et al., 1999; Garami et al., 2003). Akt also phosphorylates the transcription factor Forkhead Box class O (FoxO), a negative regulator of growth (Junger et al., 2003). In well-fed larvae, phosphorylated FoxO is excluded from the nucleus thereby allowing growth to proceed (Junger et al., 2003). In starved larvae, unphosphorylated FoxO remains in the nucleus and acts on its targets to suppress growth (Junger et al., 2003). FoxO transcriptionally regulates the translational repressor, 4E-Binding Protein (4EBP) (Miron and Sonenberg, 2001).

The IIS/TOR pathway also regulates the production of ILPs in *Drosophila*. Amino acid sensing in the fat body via the TOR pathway controls ILP synthesis and secretion in the IPCs (Britton and Edgar, 1998; Colombani et al., 2003; Géminard et al., 2009). Although the nature of the signal produced in response to amino acids is unknown, in response to dietary sugars and lipids, the fat body secretes a type I cytokine, Unpaired 2 (Upd2) (Rajan and Perrimon, 2012). In adults, Upd2 regulates ILP secretion (Rajan and Perrimon, 2012). Thus, the fat body regulates ILP secretion in response to a number of dietary macronutrients, fine-tuning body size regulation to the nutritional environment.

## Tissue-specific sensitivities to IIS/TOR signaling

**Figure 2:**
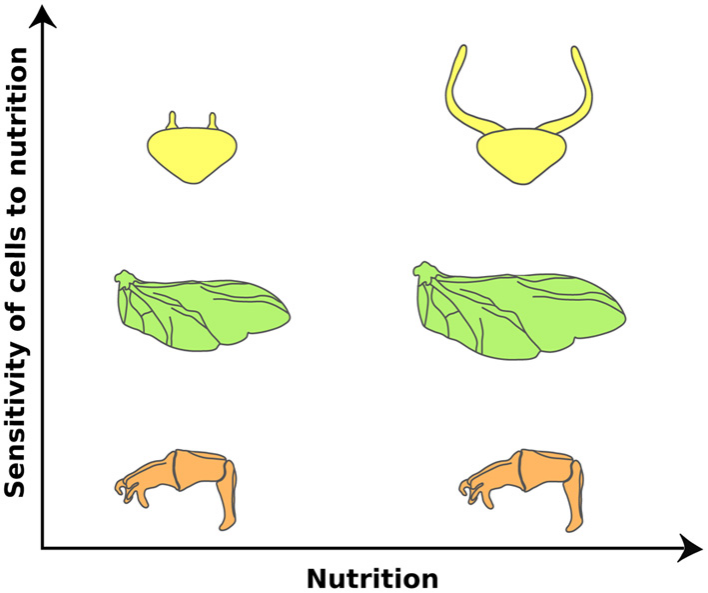
**Organs differ in their sensitivities to nutrition in the dung beetle, *Onthophagus taurus***. Nutrition affects the size of the wings (green) in proportion to the body. Other organs, such as the genital disc (orange), are less sensitive to changes in nutrition. This reduction of nutrition sensitivity is achieved by retaining high IIS/TOR signaling activity in low nutritional environments. In contrast, horn discs (yellow) show enhanced response to nutrition. Large males have disproportionately larger horns than small males. This hyperplastic response appears to be due to changes in organ sensitivity to nutrition via enhancing the IIS/TOR signaling activity. (Modified from Emlen et al., 2012).

The IIS/TOR pathway has unequal effects on the growth of different organs (Figure 2). In *Drosophila*, nutrition affects the size of the wings, palps and legs in proportion to body size through the activities of IIS/TOR signaling (Shingleton et al., 2005, 2009). Other organs are less sensitive to changes in nutrition. For instance, the size of the CNS and the male genitalia varies little with nutritional changes in body size (Shingleton et al., 2005; Cheng et al., 2011; Tang et al., 2011). The mechanisms that allow organs to become less sensitive to nutrition are presumably an adapted response to spare the effects of poor nutrition in organs where changes in size interferes with their function (Shingleton, 2010).

Organs become less sensitive to nutrition through at least two mechanisms. The CNS in *Drosophila* is protected from reductions in its size due to starvation through the activity of a “backdoor mechanism.” In starved larvae, glial cells secrete Jelly belly (Jeb), which binds to its receptor, anaplastic lymphoma kinase (Alk), and activates the IIS/TOR pathway downstream of InR (Cheng et al., 2011) (Figure 1B). In this manner, the CNS maintains its growth under starvation conditions. The genital disc uses an alternative mechanism to reduce its plasticity in response to nutrition. In *Drosophila*, the genital disc expresses low levels of FoxO mRNA and in starved larvae the genital disc shows low levels of FoxO activity (Tang et al., 2011). As a consequence, starvation has little effect on genital size (Shingleton et al., 2005, 2009; Tang et al., 2011). Overexpressing FoxO in this tissue increases its sensitivity to nutrition and results in smaller genitalia (Tang et al., 2011). Despite the differences in mechanisms between the CNS and genital disc, ultimately these organs are protected from the effects of starvation by retaining high levels of the IIS/TOR activity independent of nutritional conditions.

Other organs, such as horns in dung and rhinoceros beetles (Emlen, 1997b; Emlen et al., 2012) and mandibles in stag and broad horned flour beetles (Okada and Miyatake, 2010; Gotoh et al., 2011), show exaggerated responses to nutrition. In these organisms, large males have disproportionately larger horns or mandibles for their body size than small males. In the case of the dung and rhinoceros beetles, this hyperplastic response is caused by elevated sensitivity to changes in IIS/TOR signaling. Knocking down InR in rhinoceros beetles shows little effect on genital size, moderate effects on wing size, and more dramatic effects on horn size (Emlen et al., 2012) (Figure 2). Changing the level of activity of IIS/TOR appears to be a common mechanism for regulating the degree of plasticity in organ size. In *Drosophila*, changes in the expression of either FoxO or InR generate disproportionate growth of the wing in relation to body size (Shingleton and Tang, 2012), indicating that modulating IIS/TOR signaling at several levels of its action can produce exaggerated organ growth (Shingleton and Frankino, 2012; Shingleton and Tang, 2012).

Even in organs that scale more-or-less proportionally with body size, IIS/TOR signaling affects their growth at different points during development. In many insects, the imaginal tissues do not undergo substantial growth until after the onset of metamorphosis (Kurushima and Ohtaki, 1975; Truman et al., 2006). These organs appear less sensitive to IIS/TOR signaling in the larval feeding period (Nijhout and Grunert, 2010). Organs that grow in the larval stages can still differ in their sensitivity to nutrition at different periods of development. In *Drosophila*, the wing imaginal discs grow throughout the third (final) larval instar. Starving early third instar larvae significantly compromises wing disc growth (Shingleton et al., 2007; Mirth and Shingleton, 2012). Later in development, starvation has a much more modest effect on the growth of the wing discs (Shingleton et al., 2008). These differences in IIS/TOR sensitivity underlie differences in organ growth. Further, changes in IIS/TOR sensitivity are likely to result from cues from the metamorphic hormones.

## IIS/TOR signaling and ecdysone

The IIS/TOR pathway controls the growth of all organs, including the endocrine organs that produce hormones necessary for coordinating nutrition-dependent developmental plasticity (Hartfelder and Engels, 1998). In insects, these organs include those that synthesize the metamorphic hormones, ecdysone and JH (Hartfelder, 2000; Flatt et al., 2005; Mirth and Riddiford, 2007; Mirth and Shingleton, 2012).

Ecdysone is produced by the prothoracic glands (PGs) via a series of cytochrome P450 enzymes, the so-called Halloween genes, from a cholesterol precursor (Gilbert et al., 2002). Once synthesized, ecdysone is released from the PGs and converted to its biologically more active form, 20-hydroxyecdysone, in peripheral tissues such as fat body (Bollenbacher et al., 1977; Petryk et al., 2003). Hereafter, we refer to both forms as ecdysone.

The primary action of ecdysone is to control the larval/nymphal and metamorphic molts. Before each molt, the neuropeptide prothoracicotropic hormone (PTTH) is released to stimulate a rise in ecdysone synthesis (Truman, 1972; McBrayer et al., 2007). In the final instar of lepidopterans, coleopterans and dipterans, additional pulses of ecdysone stimulate other types of developmental transitions important for determining body size and developmental timing (Bollenbacher et al., 1981; Emlen and Nijhout, 1999; Warren et al., 2006). Many, if not all, of these pulses are regulated by environmental stimuli, such as nutrition (Emlen and Nijhout, 1999; Caldwell et al., 2005; Colombani et al., 2005; Mirth et al., 2005; Layalle et al., 2008; Gu et al., 2009; Walkiewicz and Stern, 2009; Walsh and Smith, 2011).

**Figure 3:**
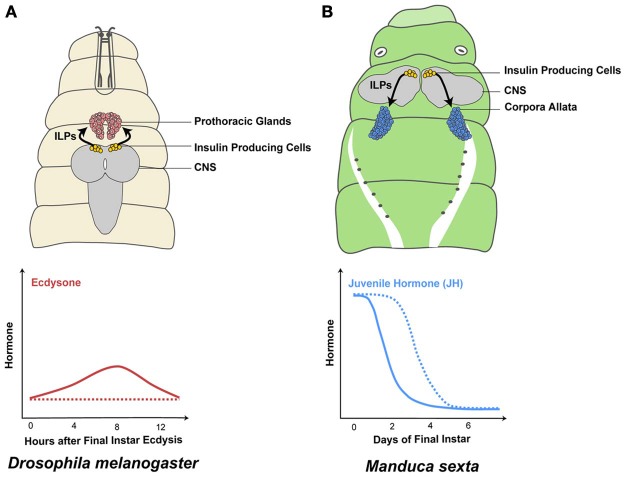
**The IIS/TOR pathway regulates metamorphic hormone synthesis**. Insulin-like peptide (ILP) secretion by the insulin producing cells in the central nervous system (CNS) depends on nutrition (Masumura et al., 2000; Brogiolo et al., 2001; Ikeya et al., 2002; Colombani et al., 2003). In well-fed final instar *Drosophila melanogaster* larvae, the prothoracic glands increase ecdysone synthesis in response to high concentrations of ILPs in the hemolymph (solid line in **A**) (Caldwell et al., 2005; Colombani et al., 2005; Mirth et al., 2005; Warren et al., 2006). In contrast, when larvae are starved early in the final instar, ecdysone synthesis is reduced/delayed (dashed line in **A**) (Caldwell et al., 2005; Colombani et al., 2005; Mirth et al., 2005). Juvenile hormone (JH) is produced in the corpora allata and the rate of JH synthesis and degradation changes in response to nutrition. Well-fed *Manduca sexta* larvae show a decrease in JH concentrations on day 1 (solid line in **B**) (Fain and Riddiford, 1975). In contrast, when *Manduca* larvae are starved at the onset of the final larval instar, JH concentrations remain high until they feed (dashed line in **B**) (Cymborowski et al., 1982).

One such pulse occurs early in the third instar of *Drosophila*. Nutrition-dependent secretion of ILPs activates the IIS/TOR pathway in the PGs and upregulates the expression of the Halloween genes *phantom* (*phm*) and *disembodied* (*dib*) (Colombani et al., 2005; Layalle et al., 2008; Walkiewicz and Stern, 2009). Repressing the IIS/TOR pathway in the PGs reduces both *phm* and *dib* transcription (Colombani et al., 2005; Layalle et al., 2008; Walkiewicz and Stern, 2009) and ecdysone concentration in early third instar larvae (Colombani et al., 2005; Mirth et al., 2005) (Figure 3A). Similarly, the IIS/TOR pathway regulates ecdysone synthesis in the final instar of other insect species, such as in the tobacco hornworm, *Manduca sexta* (Walsh and Smith, 2011; Kemirembe et al., 2012) and *Bombyx* (Gu et al., 2009). Collectively, the data outlined above indicate that the IIS/TOR pathway regulates ecdysone synthesis in response to nutrition at specific stages of development.

This stage-specific action of IIS/TOR signaling on ecdysone synthesis allows nutrition to regulate the progression and outcome of particular developmental transitions. For example, in *Drosophila*, the duration of the growth period is determined by a developmental event, known as critical weight, early in the third larval instar (Beadle et al., 1938; Caldwell et al., 2005; Colombani et al., 2005; Mirth et al., 2005; Shingleton et al., 2005). Larvae starved before reaching critical weight delay both patterning in the wing discs and the onset of metamorphosis (Beadle et al., 1938; Mirth et al., 2005; Shingleton et al., 2005; Mirth et al., 2009). Larvae starved after critical weight show normal timing in the patterning of their wing discs relative to fed larvae and initiate metamorphosis early (Beadle et al., 1938; Mirth et al., 2005; Shingleton et al., 2005; Mirth et al., 2009). Critical weight coincides with a small nutrition-sensitive ecdysone pulse known as the critical weight pulse (Warren et al., 2006; Mirth and Riddiford, 2007). By regulating ecdysone synthesis at this stage, the IIS/TOR pathway affects the progression of tissue patterning, the length of the growth period and final body size (Caldwell et al., 2005; Colombani et al., 2005; Mirth et al., 2005; Layalle et al., 2008; Mirth et al., 2009).

Ecdysone, in turn, regulates the IIS/TOR pathway throughout the body. In feeding *Drosophila* larvae, ecdysone represses TOR signaling in the fat body, which in turn produces an unknown signal that regulates ILP production in the IPCs, and hence controls systemic growth (Britton and Edgar, 1998; Colombani et al., 2003, 2005; Rusten et al., 2004; Delanoue et al., 2010). At the onset of wandering, ecdysone stimulates the production of ILP6 in the fat body to control organ growth in non-feeding stages (Okamoto et al., 2009b; Slaidina et al., 2009). Similarly, ecdysone stimulates the production of an ILP during adult development in *Bombyx* (Okamoto et al., 2009a). Thus, ecdysone can both stimulate and suppress IIS/TOR signaling in the same tissue depending on the stage.

The signaling pathway activated by ecdysone also shows several levels of interaction with the IIS/TOR pathway. Ecdysone binds to a heterodimeric nuclear hormone receptor, Ecdysone Receptor (EcR) and Ultraspiracle (Usp) (Yao et al., 1992, 1993; Talbot et al., 1993). The action of this receptor is mediated through a number of co-activators/repressors. The expression of one of the co-activators of EcR, DOR, is regulated by the IIS/TOR pathway (Francis et al., 2010). Furthermore, HR3, the product of an ecdysone response gene, regulates cell-autonomous growth through S6K activity (Montagne et al., 2010). This tightly woven net of interactions between IIS/TOR and ecdysone signaling pathways allows for fine-scale regulation of nutrition-dependent responses both between organs and across developmental stages.

## IIS/TOR signaling and JH

Considerably less is known about the pathways involved in JH signaling than for ecdysone signaling. Nevertheless, recent data have uncovered some of the mechanisms of JH action. JH determines the nature of molts by modulating the function of ecdysone. JH is known as a “*status quo*” hormone, because it prevents progression to the next life stage between molts (Riddiford, 1996). In holometabolous insects, JH concentration is high during early larval instars to prevent pupation. Only when JH concentration drops at the final larval instar do larvae undergo metamorphosis. JH is produced in the corpora allata (CA) and the removal of the CA from young larvae induces precocious metamorphosis (Williams, 1961).

Besides the *status quo* function, JH also works as a growth regulator. JH and its mimics inhibit cell proliferation in the absence of ecdysone in a lepidopteran imaginal disc-derived cell line (Oberlander et al., 2000) and in the wing discs of *Bombyx* (Koyama et al., 2004a). In the ventral diaphragm myoblasts of *Manduca*, high concentration of ecdysone suppresses cell proliferation but the same amount of ecdysone stimulates cell proliferation in the presence of JH after the wandering stage (Champlin et al., 1999), suggesting that the effects of JH on cell proliferation might be due to modulating the effects of ecdysone.

In *Drosophila*, JH binds to one of two basic helix-loop-helix Per-Arnt-Sim (bHLH-PAS) receptors with partially redundant functions: Methoprene-tolerant (Met) (Miura et al., 2005; Charles et al., 2011) and Germ cell-expressed (Gce) (Baumann et al., 2010). In other insects, JH signaling appears to occur through a single receptor, commonly referred to as Met, which appears to be the ancestral gene that duplicated to give rise to Met and Gce (Baumann et al., 2010). *Drosophila* Met null mutants are smaller than normal (Belgacem and Martin, 2007) as are animals in which the CA is ablated (Riddiford et al., 2010). Furthermore, knocking down Met produces small precocious adults in the red flour beetle, *Tribolium castaneum* (Konopova and Jindra, 2007) and the true bug, *Pyrrhocoris apterus* (Konopova et al., 2011). Thus, JH regulates body size in many insects.

The rates of JH synthesis and degradation change in response to nutrition. In *Manduca* larvae, feeding at the beginning of the final larval instar causes JH concentration to decline rapidly (Fain and Riddiford, 1975). In contrast, JH concentration remains high in starved larvae due to increased JH synthesis and to suppression of the JH degradation cascade (Cymborowski et al., 1982; Lee and Horodyski, 2006) (Figure 3B). Similarly, in adult females of *Aedes* increased IIS/TOR signaling after a blood meal causes JH levels to decline rapidly by down-regulating JH synthesis genes (Noriega, 2004; Perez-Hedo et al., 2013). Mutations in InR result in low JH concentrations in adult *Drosophila* (Tatar et al., 2001; Tu et al., 2005). Finally, knocking down FoxO suppresses starvation-dependent inhibition of JH synthesis in the German cockroach, *Blattella germanica* (Suren-Castillo et al., 2012). Therefore, IIS/TOR signaling regulates JH signaling by controlling JH synthesis and degradation in a nutrition-dependent manner.

JH, in turn, regulates the IIS/TOR pathway. Knocking down an enzyme important for JH synthesis, JH acid methyltransferase, or knocking down Met decreases ILP expression in *Tribolium* (Sheng et al., 2011). Much like ecdysone, JH both regulates and is regulated by the IIS/TOR pathway.

## Nutrition-dependent tissue plasticity in response to ecdysone and JH

Clearly, organs differ in their sensitivity to nutrition and IIS/TOR signaling, and this regulates their organ-specific growth rates. A growing body of evidence supports the hypothesis that this difference in sensitivity across organs and between stages results, in many cases, from interactions between IIS/TOR signaling and ecdysone/JH signaling pathways.

Ecdysone regulates the growth of various tissues—for example, the imaginal discs, the fat body, and the developing ovaries—in different manners (Hodin and Riddiford, 1998; Colombani et al., 2005; Mirth et al., 2009; Gancz et al., 2011). Ecdysone stimulates tissue growth and cell proliferation in the imaginal discs of *Precis coenia* (the buckeye butterfly), *Manduca*, and *Bombyx* (Champlin and Truman, 1998a,b; Nijhout and Grunert, 2002, 2010; Koyama et al., 2004a; Nijhout et al., 2007; Mirth et al., 2009). Similarly, ecdysone signaling in the *Drosophila* ovaries positively regulates ovariole number, the main determinant of ovary size (Gancz et al., 2011). In the fat body of *Drosophila*, ecdysone signaling suppresses growth by activating FoxO activity (Colombani et al., 2005; Delanoue et al., 2010). Thus, ecdysone alters growth in an organ-specific manner.

Even within the same tissue, ecdysone induces different effects depending on its concentration. Although at low concentrations ecdysone induces cell division and growth in imaginal discs, high concentrations of ecdysone tend to inhibit imaginal cell proliferation and induce differentiation (Champlin and Truman, 1998a,b; Nijhout and Grunert, 2002, 2010; Koyama et al., 2004a; Nijhout et al., 2007).

Furthermore, within the same tissue growth can rely either on IIS/TOR or ecdysone signaling depending on the stage of development. In *Drosophila*, cell divisions in the neural epithelia of the optic lobe depend on the IIS/TOR pathway early in larval development (Lanet et al., 2013). Later on in development, neural proliferation relies on ecdysone signaling, but not on IIS/TOR (Lanet et al., 2013). In the optic lobe, this ensures that proliferation necessary for the differentiation of neuronal types is protected from nutritional variation.

Finally, the effects of ecdysone on growth can depend on the nutritional context. In *Drosophila*, increasing ecdysone concentrations suppresses larval growth rate in suboptimal nutritional conditions (Colombani et al., 2005; Mirth et al., 2005). These effects on growth rate are eliminated under optimal nutritional conditions, when larvae are supplemented with live yeast. Similarly, in lepidopterans, wing imaginal discs show the greatest amount of growth in the presence of both insulin and ecdysone (Nijhout and Grunert, 2002; Nijhout et al., 2007). These data show that IIS/TOR and ecdysone signaling interact to regulate growth.

In *Manduca*, JH also regulates the growth of imaginal discs in response to nutrition. Starvation represses the growth of the wing discs, and eye and leg primordia (Macwhinnie et al., 2005; Truman et al., 2006). Eliminating JH by removing the CA partially restores disc growth even in starved larvae (Truman et al., 2006). The effects of JH on tissue growth are overridden by insulin; wing discs cultured in the presence of JH alone show reduced growth whereas when they are cultured with JH and insulin, growth rates are restored (Koyama et al., 2008).

Sensitivity to JH varies between organs. In lepidopterans, wing discs become committed to form pupal tissue shortly after the molt to the final larval instar (Ohtaki et al., 1986; Kremen and Nijhout, 1989, 1998; Obara et al., 2002; Koyama et al., 2004b). Once the discs are committed, JH no longer reverses their developmental fates. The epidermis loses sensitivity to JH much later, shortly before the onset of metamorphosis (Riddiford, 1976, 1978; Ohtaki et al., 1986; Kremen and Nijhout, 1989, 1998). This difference in timing of commitment causes organs to differ in their response to nutritional stimuli. For instance, starvation at the middle of the final instar often produces animals with larval epidermis and pupal appendages.

## A hypothesis for regulating organ plasticity

From the studies outlined above, it is clear that in insects the IIS/TOR pathway and ecdysone and JH pathways interact to control the growth of organs in a manner appropriate for the tissue and the developmental stage. For organs that show hyperplastic responses to nutrition, like beetle horns and mandibles, these mechanisms of interaction control switches for the growth of exaggerated traits.

**Figure 4:**
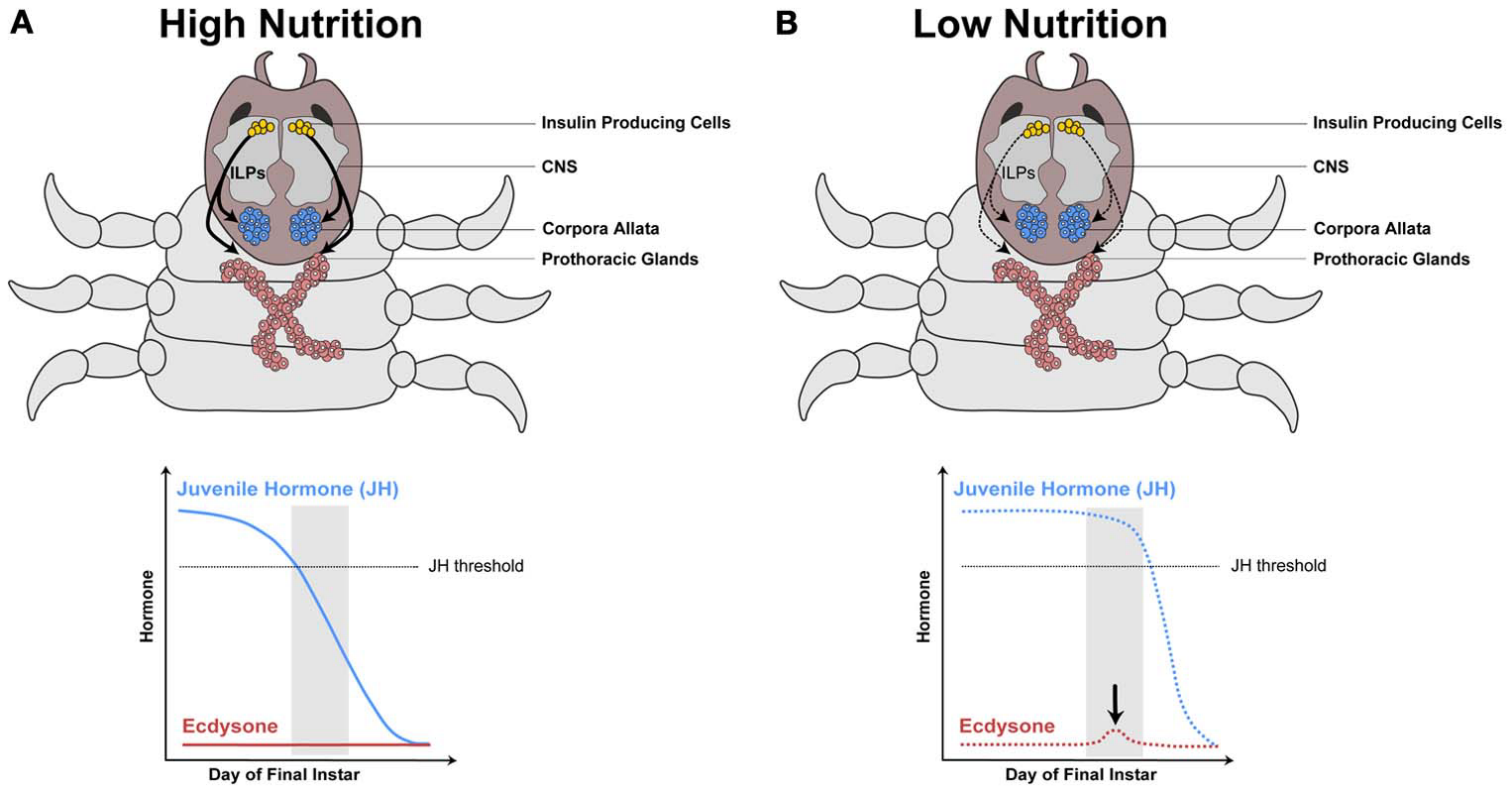
**Model hypothesizing how the IIS/TOR pathway regulates hormone synthesis in the dung beetle, *Onthophagus taurus***. In rich nutritional environments **(A)** higher concentration of insulin-like peptides (ILPs, curved black arrows) are secreted from the insulin producing cells in the central nervous system (CNS). The prothoracic glands and corpora allata grow in response to these high concentrations of ILPs, which alters the synthesis of ecdysone and juvenile hormone (JH), respectively. **(A)** In well-fed larvae, JH concentrations decline rapidly below a threshold (dashed gray line), as a consequence ecdysone does not rise during a critical stage near the end of the final larval instar (gray box). **(B)** In contrast, starved larvae have slower rates of JH decline, which triggers a small pulse of ecdysone at this stage. [Modified from Emlen and Allen (2003), Emlen et al. (2007)].

**Figure 5:**
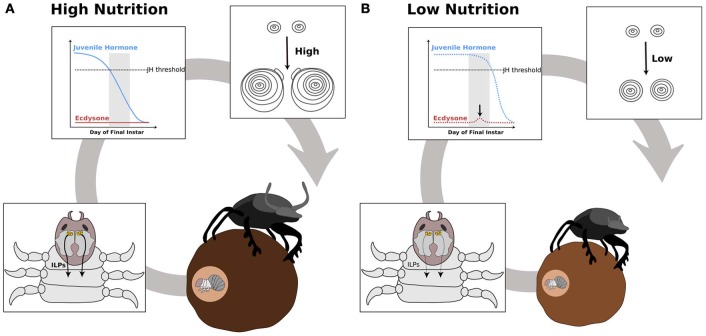
**Hypothesis outlining the role of ecdysone and juvenile hormone in switching nutrition sensitivity in the developing horns of the dung beetle, *Onthophagus taurus***. Beetle horn growth depends on larval nutrition. In well-fed larvae **(A)**, the secretion of insulin-like peptides (ILPs, curved black arrows) from the central nervous system (CNS) increases as the larvae feed (A′). At a critical stage near the end of the final instar (gray box), juvenile hormone (JH) concentration falls below threshold and ecdysone synthesis is not induced (A″). This results in high sensitivity to IIS/TOR signaling in the horn discs (A″′). As a consequence, the horn discs grow more relative to their body size (A″″). In poorly fed larvae **(B)**, ILP secretion (black curved arrows) is low (B′). Thus, JH concentration remains high during the critical stage (B″). This high level of JH appears to trigger a small pulse of ecdysone (black arrow in B″), which leads to reduced IIS/TOR sensitivity in the horn disc (B″′). The horn discs grow slowly giving rise to a small pair of horns (B″″).

We propose that nutrition, via IIS/TOR signaling, regulates ecdysone and JH synthesis, which in turn regulates IIS/TOR sensitivity in developing organs (Figure 5). For example, below a threshold body size, horn size in male dung beetles varies little with nutrition (Emlen, 1994), suggesting they are less sensitive to IIS/TOR signaling. Above the threshold body size, horns become hypersensitive to nutrition (Emlen, 1994). This developmental switch in sensitivity to nutrition between small and large males correlates with stage-dependent changes in metamorphic hormone concentration. A pulse of ecdysone occurs in the middle of the final instar in small male and female dung beetles of the species *Onthophagus taurus* (Emlen and Nijhout, 2001). Large males do not show this pulse (Emlen and Nijhout, 2001) (Figure 4). Topical application of JH at this time represses horn growth (Emlen and Nijhout, 2001), presumably by inducing the ecdysone pulse. This ecdysone pulse appears to regulate JH concentrations and/or sensitivity in later stages of development. Conversely, at the onset of metamorphosis, application of JH induces horn development in small males (Emlen and Nijhout, 1999). The exaggerated mandibles of male stag beetles, *Cyclommatus metallifer*, and horned flour beetles, *Gnatocerus cornutus*, are likewise sensitive to JH (Gotoh et al., 2011; Okada et al., 2012). Application of JH mimics induces large mandibles relative to body size for both species (Gotoh et al., 2011; Okada et al., 2012). Furthermore, in the horned flour beetle, while JH increases relative mandible size, it decreases the relative size of other structures such as the elytra and hind wings (Okada et al., 2012). This suggests that IIS/TOR signaling modifies JH and ecdysone concentrations in response to nutrition (Figure 5). These changes in metamorphic hormone concentrations alter body size and shape by reprogramming tissue-specific sensitivities to IIS/TOR to change the relative growth of tissues (Figure 5).

Social insects provide another dramatic example of how IIS/TOR signaling regulates hormone production to control body size and relative organ growth. In *Apis*, caste differentiation is determined primarily by a special nutritional component called royal jelly (Haydak, 1970; Wheeler et al., 2006; de Azevedo and Hartfelder, 2008; Wolschin et al., 2011). Larvae that are fed royal jelly differentiate into queens. If not, they develop into workers. Queens and workers differ in both size and shape: queens are larger and, because they are specialized for reproduction, their abdomens and ovaries are disproportionately larger in size compared to workers.

Royalactin, a component of royal jelly, induces queen differentiation (Kamakura, 2011) by increasing the overall concentration of JH (Rembold, 1987; Rachinsky et al., 1990). Furthermore, it induces a queen-specific JH peak in the early fifth (final) instar (Rembold, 1987; Rachinsky et al., 1990). These differences are presumably induced by accelerated growth in the CA in larvae fed royal jelly (Dogra et al., 1977), which stimulates higher rates of JH synthesis (Rachinsky and Hartfelder, 1990). Topical application of JH in larvae that are not fed royal jelly induces queen-like phenotypes (Wirtz, 1973; Nijhout and Wheeler, 1982).

Royal jelly regulates JH synthesis through the IIS/TOR pathway (Wheeler et al., 2006; de Azevedo and Hartfelder, 2008; Wolschin et al., 2011). By knocking down components of the IIS/TOR pathway to reduce its activity, the queen-specific JH pulse disappears and accordingly, the whole body of these animals becomes reprogrammed to produce worker-like phenotypes (Patel et al., 2007; Mutti et al., 2011). Topical application of JH rescues these phenotypes. Interestingly, it appears that IIS/TOR signaling regulates differential DNA methylation between queens and workers. In addition, some of these differentially methylated genes are JH-related genes (Foret et al., 2012). Finally, knocking down DNA methyltransferase induces queen-like morphology (Kucharski et al., 2008). Thus, in honeybees, a specific nutritional component induces epigenetic changes that control JH concentrations. These changes in JH reshape both whole body and relative organ growth to generate the different morphs.

## Conclusions

Over the past fifteen years, the IIS/TOR pathway has emerged as the principal pathway regulating plasticity in body size. However, the final shape of an adult insect requires both regulating overall body size and the relative growth of organs. Organs achieve specific rates of growth by differing in their sensitivities to IIS/TOR signaling both between organs and over developmental time. Further, the IIS/TOR signaling pathway regulates relative organ growth both through direct action on organs and indirectly by regulating metamorphic hormone synthesis, which have their own organ- and stage-specific effects on growth. Thus, we propose that nutrition-sensitive production of ecdysone and/or JH might act to switch organ-specific growth rates by altering their sensitivity to IIS/TOR signaling itself. This interplay between IIS/TOR and the metamorphic hormones provides a potent mechanism to dramatically alter body shape in polyphenic insects, like beetles and social insects.

### Conflict of interest statement

The authors declare that the research was conducted in the absence of any commercial or financial relationships that could be construed as a potential conflict of interest.
